# 3-Amino­benzoic acid–1,2-bis­(4-pyrid­yl)ethane (1/1)

**DOI:** 10.1107/S1600536810014261

**Published:** 2010-04-24

**Authors:** Fwu Ming Shen, Shie Fu Lush

**Affiliations:** a Department of Biotechnology, Yuanpei University, HsinChu, Taiwan 30015, People’s Republic of China; b Department of Medical Laboratory Science Biotechnology, Yuanpei University, HsinChu, Taiwan 30015, People’s Republic of China

## Abstract

The asymmetric unit of the title compound, C_12_H_12_N_2_·C_7_H_7_NO_2_, contains two 3-amino­benzoic acid mol­ecules and two 1,2-bis­(4-pyrid­yl)ethane mol­ecules. In the two 1,2-bis­(4-pyrid­yl)ethane mol­ecules, the dihedral angles between the pyridyl rings are 2.99 (9) and 46.78 (8)°. In the crystal, the mol­ecules associate through amine and carboxyl group N—H⋯O=C inter­actions between one of the 3-amino­benzoic acid mol­ecules and one of the 1,2-bis­(4-pyrid­yl)ethane mol­ecules, generating *R*_2_^2^(14) dimers, which are extended head-to-tail *via* amine and pyridine N—H⋯N hydrogen bonds. Inter­molecular O—H⋯N, N—H⋯O, N—H⋯N and C—H⋯O hydrogen bonding are observed in the crystal structure. C—H⋯π and π–π stacking inter­actions [centroid–centroid distance = 3.9985 (10) Å] are also present.

## Related literature

For applications of 3-amino­benzoic acid, see: Lynch & McClenaghan (2001 ▶); Smith (2005 ▶). For related structures, see: Smith *et al.* (1995 ▶); Lynch *et al.* (1998 ▶). For a similar dimeric 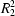
(14) structure, see: Etter *et al.* (1990 ▶).
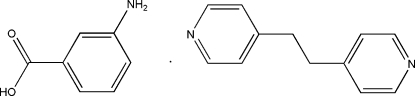


## Experimental

### 

#### Crystal data

C_12_H_12_N_2_·C_7_H_7_NO_2_
*M* *_r_* = 321.37
Triclinic, 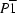

*a* = 9.0430 (3) Å
*b* = 13.0565 (5) Å
*c* = 14.6300 (5) Å
α = 88.172 (3)°
β = 79.366 (3)°
γ = 74.506 (3)°
*V* = 1635.72 (10) Å^3^
*Z* = 4
Mo *K*α radiation
μ = 0.09 mm^−1^
*T* = 100 K
0.54 × 0.18 × 0.15 mm

#### Data collection

Oxford Diffraction Gemini-S CCD diffractometer
Absorption correction: multi-scan (*CrysAlis PRO*; Oxford Diffraction, 2009 ▶) *T*_min_ = 0.997, *T*_max_ = 1.000
12385 measured reflections
5971 independent reflections
4125 reflections with *I* > 2σ(*I*)
*R*_int_ = 0.020

#### Refinement

*R*[*F* ^2^ > 2σ(*F* ^2^)] = 0.042
*wR*(*F* ^2^) = 0.108
*S* = 0.99
5971 reflections
455 parameters
2 restraints
H atoms treated by a mixture of independent and constrained refinement
Δρ_max_ = 0.57 e Å^−3^
Δρ_min_ = −0.35 e Å^−3^

### 

Data collection: *CrysAlis CCD* (Oxford Diffraction, 2008 ▶); cell refinement: *CrysAlis RED* (Oxford Diffraction, 2008 ▶); data reduction: *CrysAlis RED*; program(s) used to solve structure: *SHELXS97* (Sheldrick, 2008 ▶); program(s) used to refine structure: *SHELXL97* (Sheldrick, 2008 ▶); molecular graphics: *PLATON* (Spek, 2009 ▶); software used to prepare material for publication: *PLATON*.

## Supplementary Material

Crystal structure: contains datablocks I, global. DOI: 10.1107/S1600536810014261/xu2742sup1.cif

Structure factors: contains datablocks I. DOI: 10.1107/S1600536810014261/xu2742Isup2.hkl

Additional supplementary materials:  crystallographic information; 3D view; checkCIF report

## Figures and Tables

**Table 1:** Hydrogen-bond geometry (Å, °) *Cg*5 is the centroid of the C2–C7 ring.

*D*—H⋯*A*	*D*—H	H⋯*A*	*D*⋯*A*	*D*—H⋯*A*
O1—H1*C*⋯N2	0.84 (2)	1.79 (2)	2.6294 (19)	176 (2)
O3—H3*A*⋯N6^i^	0.84 (1)	1.75 (1)	2.5790 (19)	171 (2)
N1—H1*A*⋯O4^ii^	0.89 (2)	2.21 (2)	3.061 (2)	158.6 (17)
N1—H1*B*⋯N3^iii^	0.882 (19)	2.17 (2)	3.048 (2)	177.8 (19)
N4—H4*A*⋯O2^ii^	0.89 (2)	2.19 (2)	3.035 (2)	157.6 (18)
N4—H4*B*⋯N5^iv^	0.89 (2)	2.13 (2)	3.017 (2)	171.9 (17)
C3—H3*C*⋯O4^ii^	0.95	2.56	3.353 (2)	141
C22—H22*A*⋯O2^ii^	0.95	2.54	3.327 (2)	141
C28—H28*A*⋯O2^v^	0.95	2.55	3.492 (2)	172
C38—H38*A*⋯O4^vi^	0.95	2.50	3.448 (2)	177
C12—H12*A*⋯*Cg*5^v^	0.95	2.67	3.5510 (18)	154

Symmetry codes: (i) 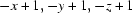; (ii) 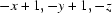; (iii) 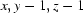; (iv) 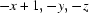; (v) 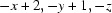; (vi) 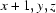.

**Fig. 1.**
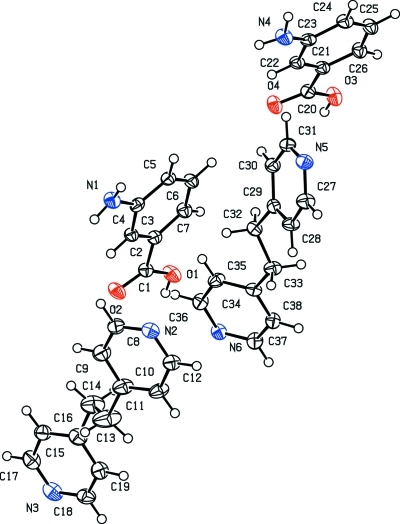
View of the title compound with the atom numbering scheme. Displacement ellipsoids for non-H atoms are drawn at the 50% probability level.

**Fig. 2.**
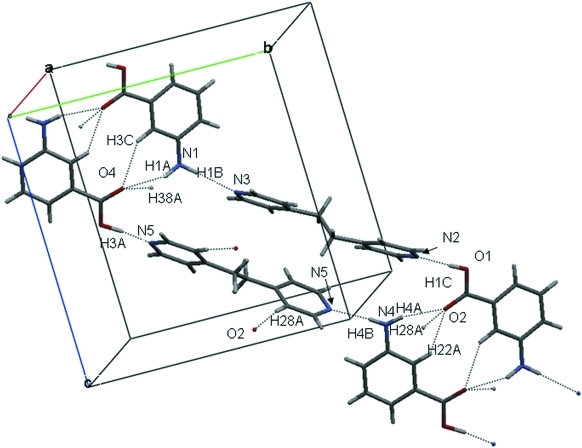
The molecular packing for the title compound. Hydrogen-bonding associations are shown as dotted lines.

## supplementary crystallographic information

### Comment

The structures of a number of 1:1 adduct compounds of 3-aminobenzoic acid with Lewis bases have been reported. These include compounds with 2-amino-pyrimidine (Smith *et al.*, 1995) and 2-aminobenzothiazole (Lynch *et al.*, 1998). Not only 3-aminobenzoic acid is more effective for the promotion of hydrogen-bonding extensions, but also forms various weak noncovalent interactions, such as π-π stacking and C—H···π interactions.

Our research program aims to gain hydrogen-bonding networks involving 3-aminobenzoic acid with 1,2-bis(4-pyridyl)ethane, which can develop well defined noncovalent supramolecular architectures and form multiple hydrogen bonds containing components of complementary arrays of hydrogen-bonding sites. The resulting crystal X-Ray structure (Fig. 1) consists entirely of neutral 3-aminobenzoic acid molecules and neutral 1,2-bis(4-pyridyl)ethane units, with no proton transfer (Lynch & McClenaghan, 2001; Smith, 2005). The title compound comprises non-planar molecules, similar to other analogous compounds (Smith *et al.*, 1995; Lynch *et al.*, 1998) that associate 3-aminobenzoic acid via amine and carboxylic group N—H···O [N···O 3.061 (2) and 3.035 (2) Å] dimer R_2_^2^(14) (Etter *et al.*, 1990) and form linear hydrogen-bonded via amine and pyridine N—H··· N [N···N 3.048 (2) and 3.017 (2) Å].

The title compound's supramolecular structure can be readily analyzed in terms of carboxyl atom O1 and amino group N1 act as hydrogen-bond donors to pyridyl atoms N2 and N3. Similarly, N4, C22, C28, N1, C3, C38 act as hydrogen-bond donors to carboxyl atoms O2, O4, respectively (Table 1 and Fig. 2).

This layer is consolidated by C—H ···π interaction (C12—H12A···Cg5; full details and symmetry code are given in Table 1). Futhermore, π-π ring stacking interactions are between neighboring complexes in the structure. The distance between Cg1 (N2/C8—C12)···Cg4 (N6/C34—C38) is 3.9985 (10) Å and dihedral angle between two rings is 9.86 (9) °.

### Experimental

The 3-aminobenzoic acid (171.0 mg, 1.0 mmol) and 1,2-bis(4-pyridyl)ethane (184 mg, 1.0 mmol) were dissolved in 20 ml 50% methanol-water, the solution was refluxed for 30 min. The filtered solution was transferred to a 25 ml tube after one week at room temperature, and colorless transparent crystals formed (yield 59.36%). Elemental analysis calcd(%) for C_19_H_19_N_3_O_2_ (Mr=321.37): C, 55.83; H, 5.96; N, 13.08; Found: C, 56.03; H, 5.92; N, 12.96.

### Refinement

Amino H atoms were located in a difference Fourier map and were refined isotropically. Water H atoms were located in a difference Fourier map and refined with the distances constraints of O—H = 0.84 Å, U_iso_(H) = 1.5U_eq_(O). Other H atoms were positioned geometrically with C—H = 0.95 (aromatic) and 0.99 Å (methylene), and refined using a riding model with U_iso_(H) = 1.2U_eq_(C).

### Crystal data

**Table:**

C_12_H_12_N_2_·C_7_H_7_NO_2_	*Z* = 4
*M**_r_* = 321.37	*F*(000) = 680
Triclinic, *P*1	*D*_x_ = 1.305 Mg m^−^^3^
Hall symbol: -P 1	Mo *K*α radiation, λ = 0.71073 Å
*a* = 9.0430 (3) Å	Cell parameters from 6302 reflections
*b* = 13.0565 (5) Å	θ = 2.5–29.2°
*c* = 14.6300 (5) Å	µ = 0.09 mm^−^^1^
α = 88.172 (3)°	*T* = 100 K
β = 79.366 (3)°	Parallelepiped, colorless
γ = 74.506 (3)°	0.54 × 0.18 × 0.15 mm
*V* = 1635.72 (10) Å^3^	

### Data collection

**Table:**

Oxford Diffraction Gemini-S CCD diffractometer	5971 independent reflections
Radiation source: fine-focus sealed tube	4125 reflections with *I* > 2σ(*I*)
graphite	*R*_int_ = 0.020
ω scans	θ_max_ = 25.5°, θ_min_ = 2.5°
Absorption correction: multi-scan (*CrysAlis PRO*; Oxford Diffraction, 2009)	*h* = −10→10
*T*_min_ = 0.997, *T*_max_ = 1.000	*k* = −13→15
12385 measured reflections	*l* = −17→14

### Refinement

**Table:**

Refinement on *F*^2^	Primary atom site location: structure-invariant direct methods
Least-squares matrix: full	Secondary atom site location: difference Fourier map
*R*[*F*^2^ > 2σ(*F*^2^)] = 0.042	Hydrogen site location: inferred from neighbouring sites
*wR*(*F*^2^) = 0.108	H atoms treated by a mixture of independent and constrained refinement
*S* = 0.99	*w* = 1/[σ^2^(*F*_o_^2^) + (0.0632*P*)^2^] where *P* = (*F*_o_^2^ + 2*F*_c_^2^)/3
5971 reflections	(Δ/σ)_max_ = 0.002
455 parameters	Δρ_max_ = 0.57 e Å^−^^3^
2 restraints	Δρ_min_ = −0.35 e Å^−^^3^

### Special details

**Table:**

Geometry. All esds (except the esd in the dihedral angle between two l.s. planes) are estimated using the full covariance matrix. The cell esds are taken into account individually in the estimation of esds in distances, angles and torsion angles; correlations between esds in cell parameters are only used when they are defined by crystal symmetry. An approximate (isotropic) treatment of cell esds is used for estimating esds involving l.s. planes.

### Fractional atomic coordinates and isotropic or equivalent isotropic displacement parameters (Å^2^)

**Table:**

	*x*	*y*	*z*	*U*_iso_*/*U*_eq_	
O1	0.76671 (15)	0.58027 (10)	0.03742 (8)	0.0334 (3)	
O2	0.77042 (15)	0.69496 (9)	−0.07886 (8)	0.0328 (3)	
O3	0.26995 (15)	0.16295 (9)	0.48714 (9)	0.0323 (3)	
O4	0.23015 (14)	0.28364 (9)	0.37667 (8)	0.0312 (3)	
N1	0.69956 (18)	0.49939 (14)	−0.35303 (11)	0.0306 (4)	
N2	0.81019 (17)	0.73452 (11)	0.13170 (10)	0.0281 (3)	
N3	0.7482 (2)	1.31954 (12)	0.50754 (11)	0.0374 (4)	
N4	0.28039 (18)	0.06579 (14)	0.08726 (11)	0.0305 (4)	
N5	0.77629 (17)	0.10653 (11)	0.02344 (10)	0.0299 (4)	
N6	0.76549 (16)	0.67214 (11)	0.41027 (10)	0.0261 (3)	
C1	0.75649 (18)	0.60925 (13)	−0.04865 (12)	0.0230 (4)	
C2	0.72593 (18)	0.52804 (13)	−0.10732 (12)	0.0225 (4)	
C3	0.73369 (18)	0.54740 (13)	−0.20172 (12)	0.0226 (4)	
H3C	0.7591	0.6100	−0.2264	0.027*	
C4	0.70458 (18)	0.47598 (13)	−0.26121 (12)	0.0238 (4)	
C5	0.67203 (19)	0.38323 (13)	−0.22244 (13)	0.0254 (4)	
H5B	0.6563	0.3319	−0.2615	0.030*	
C6	0.66242 (19)	0.36513 (13)	−0.12816 (13)	0.0277 (4)	
H6B	0.6380	0.3023	−0.1033	0.033*	
C7	0.68795 (19)	0.43750 (13)	−0.06922 (13)	0.0264 (4)	
H7A	0.6796	0.4253	−0.0043	0.032*	
C8	0.7001 (2)	0.82631 (14)	0.13950 (13)	0.0314 (4)	
H8A	0.6139	0.8324	0.1092	0.038*	
C9	0.7045 (2)	0.91298 (15)	0.18921 (14)	0.0368 (5)	
H9A	0.6211	0.9760	0.1947	0.044*	
C10	0.8319 (2)	0.90730 (15)	0.23106 (14)	0.0384 (5)	
C11	0.9444 (2)	0.81133 (15)	0.22388 (13)	0.0353 (5)	
H11A	1.0322	0.8031	0.2532	0.042*	
C12	0.9304 (2)	0.72758 (14)	0.17472 (13)	0.0297 (4)	
H12A	1.0092	0.6623	0.1712	0.036*	
C13	0.8492 (2)	1.00327 (19)	0.28015 (19)	0.0631 (7)	
H13A	0.9396	0.9800	0.3122	0.076*	
H13B	0.8729	1.0551	0.2326	0.076*	
C14	0.7126 (2)	1.05708 (17)	0.34813 (16)	0.0475 (6)	
H14A	0.6866	1.0047	0.3945	0.057*	
H14B	0.6229	1.0826	0.3157	0.057*	
C15	0.7326 (2)	1.15073 (15)	0.39924 (13)	0.0335 (5)	
C16	0.6186 (2)	1.24545 (14)	0.40946 (13)	0.0326 (4)	
H16A	0.5313	1.2550	0.3795	0.039*	
C17	0.6305 (2)	1.32601 (15)	0.46274 (14)	0.0363 (5)	
H17A	0.5499	1.3906	0.4681	0.044*	
C18	0.8615 (2)	1.22878 (15)	0.49533 (13)	0.0341 (5)	
H18A	0.9484	1.2223	0.5251	0.041*	
C19	0.8598 (2)	1.14407 (15)	0.44223 (13)	0.0341 (5)	
H19A	0.9445	1.0818	0.4350	0.041*	
C20	0.25604 (19)	0.19120 (13)	0.40181 (12)	0.0242 (4)	
C21	0.27294 (18)	0.10109 (13)	0.33654 (12)	0.0228 (4)	
C22	0.26585 (18)	0.12404 (13)	0.24435 (12)	0.0232 (4)	
H22A	0.2535	0.1952	0.2242	0.028*	
C23	0.27665 (18)	0.04385 (13)	0.18016 (12)	0.0235 (4)	
C24	0.28837 (19)	−0.05921 (13)	0.21297 (13)	0.0270 (4)	
H24A	0.2917	−0.1145	0.1714	0.032*	
C25	0.2952 (2)	−0.08156 (14)	0.30540 (13)	0.0301 (4)	
H25A	0.3038	−0.1522	0.3263	0.036*	
C26	0.28984 (19)	−0.00229 (13)	0.36817 (13)	0.0268 (4)	
H26A	0.2975	−0.0184	0.4312	0.032*	
C27	0.8959 (2)	0.14566 (14)	0.03144 (13)	0.0313 (4)	
H27A	0.9958	0.1120	−0.0034	0.038*	
C28	0.8827 (2)	0.23233 (13)	0.08754 (12)	0.0280 (4)	
H28A	0.9720	0.2566	0.0904	0.034*	
C29	0.73906 (19)	0.28338 (13)	0.13937 (12)	0.0227 (4)	
C30	0.6147 (2)	0.24242 (13)	0.13143 (12)	0.0264 (4)	
H30A	0.5137	0.2739	0.1661	0.032*	
C31	0.6377 (2)	0.15652 (14)	0.07347 (13)	0.0284 (4)	
H31A	0.5501	0.1312	0.0687	0.034*	
C32	0.71112 (19)	0.38225 (13)	0.19731 (12)	0.0243 (4)	
H32A	0.6204	0.3855	0.2477	0.029*	
H32B	0.6829	0.4448	0.1577	0.029*	
C33	0.84736 (19)	0.39114 (13)	0.24075 (13)	0.0282 (4)	
H33A	0.8779	0.3278	0.2790	0.034*	
H33B	0.9373	0.3905	0.1904	0.034*	
C34	0.81443 (18)	0.48966 (13)	0.30095 (12)	0.0230 (4)	
C35	0.69101 (19)	0.57861 (13)	0.29678 (12)	0.0280 (4)	
H35A	0.6204	0.5786	0.2560	0.034*	
C36	0.6707 (2)	0.66686 (13)	0.35164 (12)	0.0277 (4)	
H36A	0.5854	0.7267	0.3476	0.033*	
C37	0.8843 (2)	0.58632 (13)	0.41499 (12)	0.0287 (4)	
H37A	0.9527	0.5884	0.4565	0.034*	
C38	0.9119 (2)	0.49513 (13)	0.36243 (12)	0.0265 (4)	
H38A	0.9975	0.4361	0.3683	0.032*	
H1C	0.785 (2)	0.6290 (10)	0.0663 (12)	0.040*	
H3A	0.268 (2)	0.2174 (9)	0.5172 (12)	0.040*	
H1A	0.731 (2)	0.5562 (16)	−0.3754 (13)	0.034 (5)*	
H1B	0.714 (2)	0.4461 (15)	−0.3921 (14)	0.036 (5)*	
H4A	0.252 (2)	0.1339 (18)	0.0720 (15)	0.051 (7)*	
H4B	0.256 (2)	0.0199 (16)	0.0528 (14)	0.044 (6)*	

### Atomic displacement parameters (Å^2^)

**Table:**

	*U*^11^	*U*^22^	*U*^33^	*U*^12^	*U*^13^	*U*^23^
O1	0.0541 (8)	0.0295 (7)	0.0231 (8)	−0.0198 (6)	−0.0098 (6)	−0.0049 (6)
O2	0.0516 (8)	0.0259 (7)	0.0270 (7)	−0.0185 (6)	−0.0100 (6)	−0.0021 (6)
O3	0.0530 (8)	0.0223 (7)	0.0236 (8)	−0.0093 (6)	−0.0118 (6)	−0.0049 (6)
O4	0.0489 (8)	0.0192 (7)	0.0268 (7)	−0.0078 (6)	−0.0110 (6)	−0.0042 (5)
N1	0.0430 (9)	0.0253 (9)	0.0265 (10)	−0.0107 (8)	−0.0106 (7)	−0.0061 (8)
N2	0.0338 (8)	0.0304 (9)	0.0207 (8)	−0.0117 (7)	−0.0011 (7)	−0.0057 (7)
N3	0.0517 (10)	0.0340 (10)	0.0277 (9)	−0.0169 (8)	−0.0006 (8)	−0.0077 (7)
N4	0.0430 (10)	0.0251 (9)	0.0262 (10)	−0.0120 (8)	−0.0078 (7)	−0.0087 (8)
N5	0.0357 (9)	0.0276 (8)	0.0292 (9)	−0.0113 (7)	−0.0076 (7)	−0.0067 (7)
N6	0.0342 (8)	0.0240 (8)	0.0203 (8)	−0.0078 (6)	−0.0046 (7)	−0.0050 (6)
C1	0.0234 (8)	0.0217 (9)	0.0228 (10)	−0.0052 (7)	−0.0018 (7)	−0.0050 (8)
C2	0.0211 (8)	0.0202 (9)	0.0251 (10)	−0.0032 (7)	−0.0035 (7)	−0.0061 (7)
C3	0.0231 (8)	0.0177 (9)	0.0267 (10)	−0.0047 (7)	−0.0039 (7)	−0.0047 (7)
C4	0.0199 (8)	0.0225 (9)	0.0273 (11)	−0.0005 (7)	−0.0064 (7)	−0.0072 (8)
C5	0.0243 (9)	0.0178 (9)	0.0350 (12)	−0.0030 (7)	−0.0102 (8)	−0.0093 (8)
C6	0.0287 (9)	0.0188 (9)	0.0369 (12)	−0.0075 (7)	−0.0069 (8)	−0.0024 (8)
C7	0.0296 (9)	0.0234 (10)	0.0262 (10)	−0.0067 (7)	−0.0047 (8)	−0.0034 (8)
C8	0.0345 (10)	0.0334 (11)	0.0296 (11)	−0.0109 (8)	−0.0112 (8)	−0.0020 (9)
C9	0.0321 (10)	0.0301 (11)	0.0476 (13)	−0.0047 (8)	−0.0093 (9)	−0.0105 (9)
C10	0.0310 (10)	0.0368 (11)	0.0473 (13)	−0.0073 (8)	−0.0060 (9)	−0.0197 (10)
C11	0.0265 (9)	0.0409 (12)	0.0397 (12)	−0.0073 (8)	−0.0089 (9)	−0.0146 (9)
C12	0.0265 (9)	0.0288 (10)	0.0325 (11)	−0.0063 (8)	−0.0015 (8)	−0.0097 (8)
C13	0.0382 (12)	0.0631 (16)	0.087 (2)	−0.0124 (11)	−0.0032 (12)	−0.0452 (14)
C14	0.0461 (12)	0.0530 (14)	0.0454 (14)	−0.0181 (11)	−0.0027 (11)	−0.0187 (11)
C15	0.0413 (11)	0.0320 (11)	0.0293 (11)	−0.0122 (9)	−0.0067 (9)	−0.0071 (9)
C16	0.0329 (10)	0.0339 (11)	0.0343 (12)	−0.0131 (8)	−0.0084 (8)	0.0014 (9)
C17	0.0359 (10)	0.0294 (11)	0.0380 (12)	−0.0060 (8)	0.0044 (9)	−0.0039 (9)
C18	0.0396 (11)	0.0377 (11)	0.0309 (11)	−0.0179 (9)	−0.0094 (9)	−0.0028 (9)
C19	0.0353 (10)	0.0295 (10)	0.0356 (12)	−0.0058 (8)	−0.0044 (9)	−0.0064 (9)
C20	0.0270 (9)	0.0228 (10)	0.0233 (11)	−0.0065 (7)	−0.0057 (8)	−0.0042 (8)
C21	0.0221 (8)	0.0208 (9)	0.0260 (10)	−0.0063 (7)	−0.0039 (7)	−0.0051 (7)
C22	0.0228 (8)	0.0205 (9)	0.0272 (10)	−0.0070 (7)	−0.0040 (7)	−0.0056 (7)
C23	0.0204 (8)	0.0242 (10)	0.0263 (11)	−0.0069 (7)	−0.0029 (7)	−0.0079 (8)
C24	0.0271 (9)	0.0225 (9)	0.0316 (11)	−0.0065 (7)	−0.0039 (8)	−0.0116 (8)
C25	0.0324 (10)	0.0196 (9)	0.0381 (12)	−0.0068 (8)	−0.0055 (8)	−0.0037 (8)
C26	0.0313 (9)	0.0223 (9)	0.0267 (11)	−0.0060 (7)	−0.0061 (8)	−0.0023 (8)
C27	0.0312 (10)	0.0315 (10)	0.0312 (11)	−0.0091 (8)	−0.0024 (8)	−0.0111 (8)
C28	0.0272 (9)	0.0282 (10)	0.0316 (11)	−0.0114 (7)	−0.0052 (8)	−0.0075 (8)
C29	0.0295 (9)	0.0205 (9)	0.0199 (10)	−0.0071 (7)	−0.0082 (7)	0.0002 (7)
C30	0.0263 (9)	0.0247 (9)	0.0286 (11)	−0.0068 (7)	−0.0056 (8)	−0.0025 (8)
C31	0.0293 (9)	0.0279 (10)	0.0326 (11)	−0.0114 (8)	−0.0115 (8)	−0.0018 (8)
C32	0.0284 (9)	0.0216 (9)	0.0229 (10)	−0.0066 (7)	−0.0034 (7)	−0.0050 (7)
C33	0.0260 (9)	0.0270 (10)	0.0314 (11)	−0.0070 (7)	−0.0027 (8)	−0.0125 (8)
C34	0.0237 (9)	0.0251 (9)	0.0208 (10)	−0.0098 (7)	0.0004 (7)	−0.0050 (7)
C35	0.0257 (9)	0.0300 (10)	0.0296 (11)	−0.0063 (7)	−0.0080 (8)	−0.0088 (8)
C36	0.0298 (9)	0.0257 (10)	0.0256 (10)	−0.0029 (7)	−0.0055 (8)	−0.0061 (8)
C37	0.0374 (10)	0.0281 (10)	0.0218 (10)	−0.0069 (8)	−0.0101 (8)	−0.0050 (8)
C38	0.0302 (9)	0.0242 (9)	0.0242 (10)	−0.0048 (7)	−0.0058 (8)	−0.0028 (8)

### Geometric parameters (Å, °)

**Table:**

O1—C1	1.316 (2)	C14—H14A	0.9900
O1—H1C	0.843 (16)	C14—H14B	0.9900
O2—C1	1.2192 (19)	C15—C16	1.374 (3)
O3—C20	1.309 (2)	C15—C19	1.391 (2)
O3—H3A	0.842 (14)	C16—C17	1.367 (2)
O4—C20	1.2245 (19)	C16—H16A	0.9500
N1—C4	1.374 (2)	C17—H17A	0.9500
N1—H1A	0.89 (2)	C18—C19	1.377 (2)
N1—H1B	0.881 (19)	C18—H18A	0.9500
N2—C8	1.330 (2)	C19—H19A	0.9500
N2—C12	1.335 (2)	C20—C21	1.497 (2)
N3—C17	1.332 (2)	C21—C22	1.382 (2)
N3—C18	1.335 (2)	C21—C26	1.391 (2)
N4—C23	1.376 (2)	C22—C23	1.401 (2)
N4—H4A	0.89 (2)	C22—H22A	0.9500
N4—H4B	0.889 (19)	C23—C24	1.398 (2)
N5—C31	1.337 (2)	C24—C25	1.383 (3)
N5—C27	1.338 (2)	C24—H24A	0.9500
N6—C36	1.335 (2)	C25—C26	1.391 (2)
N6—C37	1.339 (2)	C25—H25A	0.9500
C1—C2	1.497 (2)	C26—H26A	0.9500
C2—C3	1.388 (2)	C27—C28	1.386 (2)
C2—C7	1.389 (2)	C27—H27A	0.9500
C3—C4	1.401 (2)	C28—C29	1.383 (2)
C3—H3C	0.9500	C28—H28A	0.9500
C4—C5	1.400 (2)	C29—C30	1.392 (2)
C5—C6	1.383 (3)	C29—C32	1.505 (2)
C5—H5B	0.9500	C30—C31	1.375 (2)
C6—C7	1.390 (2)	C30—H30A	0.9500
C6—H6B	0.9500	C31—H31A	0.9500
C7—H7A	0.9500	C32—C33	1.518 (2)
C8—C9	1.378 (2)	C32—H32A	0.9900
C8—H8A	0.9500	C32—H32B	0.9900
C9—C10	1.385 (2)	C33—C34	1.513 (2)
C9—H9A	0.9500	C33—H33A	0.9900
C10—C11	1.380 (3)	C33—H33B	0.9900
C10—C13	1.521 (3)	C34—C38	1.386 (2)
C11—C12	1.374 (2)	C34—C35	1.388 (2)
C11—H11A	0.9500	C35—C36	1.377 (2)
C12—H12A	0.9500	C35—H35A	0.9500
C13—C14	1.462 (3)	C36—H36A	0.9500
C13—H13A	0.9900	C37—C38	1.378 (2)
C13—H13B	0.9900	C37—H37A	0.9500
C14—C15	1.521 (3)	C38—H38A	0.9500
			
C1—O1—H1C	109.6 (14)	N3—C18—C19	123.90 (17)
C20—O3—H3A	107.9 (14)	N3—C18—H18A	118.1
C4—N1—H1A	117.4 (12)	C19—C18—H18A	118.1
C4—N1—H1B	117.8 (12)	C18—C19—C15	119.26 (17)
H1A—N1—H1B	117.4 (18)	C18—C19—H19A	120.4
C8—N2—C12	117.35 (14)	C15—C19—H19A	120.4
C17—N3—C18	115.91 (15)	O4—C20—O3	123.30 (15)
C23—N4—H4A	117.2 (14)	O4—C20—C21	122.09 (15)
C23—N4—H4B	117.1 (13)	O3—C20—C21	114.61 (15)
H4A—N4—H4B	116.4 (19)	C22—C21—C26	120.79 (15)
C31—N5—C27	116.04 (14)	C22—C21—C20	118.06 (15)
C36—N6—C37	117.17 (14)	C26—C21—C20	121.11 (15)
O2—C1—O1	123.33 (15)	C21—C22—C23	120.95 (16)
O2—C1—C2	122.23 (16)	C21—C22—H22A	119.5
O1—C1—C2	114.44 (15)	C23—C22—H22A	119.5
C3—C2—C7	120.77 (15)	N4—C23—C24	121.30 (15)
C3—C2—C1	117.48 (15)	N4—C23—C22	120.70 (16)
C7—C2—C1	121.74 (16)	C24—C23—C22	117.97 (16)
C2—C3—C4	121.01 (16)	C25—C24—C23	120.70 (15)
C2—C3—H3C	119.5	C25—C24—H24A	119.7
C4—C3—H3C	119.5	C23—C24—H24A	119.7
N1—C4—C5	121.39 (15)	C24—C25—C26	121.05 (17)
N1—C4—C3	120.93 (16)	C24—C25—H25A	119.5
C5—C4—C3	117.57 (16)	C26—C25—H25A	119.5
C6—C5—C4	121.08 (15)	C25—C26—C21	118.46 (16)
C6—C5—H5B	119.5	C25—C26—H26A	120.8
C4—C5—H5B	119.5	C21—C26—H26A	120.8
C5—C6—C7	120.99 (17)	N5—C27—C28	123.95 (17)
C5—C6—H6B	119.5	N5—C27—H27A	118.0
C7—C6—H6B	119.5	C28—C27—H27A	118.0
C2—C7—C6	118.52 (17)	C29—C28—C27	119.67 (15)
C2—C7—H7A	120.7	C29—C28—H28A	120.2
C6—C7—H7A	120.7	C27—C28—H28A	120.2
N2—C8—C9	123.44 (16)	C28—C29—C30	116.43 (15)
N2—C8—H8A	118.3	C28—C29—C32	123.50 (14)
C9—C8—H8A	118.3	C30—C29—C32	119.95 (15)
C8—C9—C10	119.32 (17)	C31—C30—C29	120.15 (16)
C8—C9—H9A	120.3	C31—C30—H30A	119.9
C10—C9—H9A	120.3	C29—C30—H30A	119.9
C11—C10—C9	116.86 (16)	N5—C31—C30	123.75 (15)
C11—C10—C13	121.56 (17)	N5—C31—H31A	118.1
C9—C10—C13	121.56 (18)	C30—C31—H31A	118.1
C12—C11—C10	120.48 (16)	C29—C32—C33	115.58 (14)
C12—C11—H11A	119.8	C29—C32—H32A	108.4
C10—C11—H11A	119.8	C33—C32—H32A	108.4
N2—C12—C11	122.48 (16)	C29—C32—H32B	108.4
N2—C12—H12A	118.8	C33—C32—H32B	108.4
C11—C12—H12A	118.8	H32A—C32—H32B	107.4
C14—C13—C10	114.94 (18)	C34—C33—C32	114.45 (14)
C14—C13—H13A	108.5	C34—C33—H33A	108.6
C10—C13—H13A	108.5	C32—C33—H33A	108.6
C14—C13—H13B	108.5	C34—C33—H33B	108.6
C10—C13—H13B	108.5	C32—C33—H33B	108.6
H13A—C13—H13B	107.5	H33A—C33—H33B	107.6
C13—C14—C15	114.66 (17)	C38—C34—C35	116.73 (15)
C13—C14—H14A	108.6	C38—C34—C33	120.09 (15)
C15—C14—H14A	108.6	C35—C34—C33	123.16 (14)
C13—C14—H14B	108.6	C36—C35—C34	120.06 (15)
C15—C14—H14B	108.6	C36—C35—H35A	120.0
H14A—C14—H14B	107.6	C34—C35—H35A	120.0
C16—C15—C19	116.69 (16)	N6—C36—C35	123.04 (16)
C16—C15—C14	120.43 (17)	N6—C36—H36A	118.5
C19—C15—C14	122.78 (17)	C35—C36—H36A	118.5
C17—C16—C15	120.10 (17)	N6—C37—C38	123.11 (15)
C17—C16—H16A	119.9	N6—C37—H37A	118.4
C15—C16—H16A	119.9	C38—C37—H37A	118.4
N3—C17—C16	124.04 (18)	C37—C38—C34	119.88 (16)
N3—C17—H17A	118.0	C37—C38—H38A	120.1
C16—C17—H17A	118.0	C34—C38—H38A	120.1

### Hydrogen-bond geometry (Å, °)

**Table:**

Cg5 is the centroid of the C2–C7 ring.

**Table:**

*D*—H···*A*	*D*—H	H···*A*	*D*···*A*	*D*—H···*A*
O1—H1C···N2	0.84 (2)	1.79 (2)	2.6294 (19)	176 (2)
O3—H3A···N6^i^	0.84 (1)	1.75 (1)	2.5790 (19)	171 (2)
N1—H1A···O4^ii^	0.89 (2)	2.21 (2)	3.061 (2)	158.6 (17)
N1—H1B···N3^iii^	0.882 (19)	2.17 (2)	3.048 (2)	177.8 (19)
N4—H4A···O2^ii^	0.89 (2)	2.19 (2)	3.035 (2)	157.6 (18)
N4—H4B···N5^iv^	0.89 (2)	2.13 (2)	3.017 (2)	171.9 (17)
C3—H3C···O4^ii^	0.95	2.56	3.353 (2)	141
C22—H22A···O2^ii^	0.95	2.54	3.327 (2)	141
C28—H28A···O2^v^	0.95	2.55	3.492 (2)	172
C38—H38A···O4^vi^	0.95	2.50	3.448 (2)	177
C12—H12A···Cg5^v^	0.95	2.67	3.5510 (18)	154

Symmetry codes: (i) −*x*+1, −*y*+1, −*z*+1; (ii) −*x*+1, −*y*+1, −*z*; (iii) *x*, *y*−1, *z*−1; (iv) −*x*+1, −*y*, −*z*; (v) −*x*+2, −*y*+1, −*z*; (vi) *x*+1, *y*, *z*.
